# Characterization of Cultivar Differences of Blueberry Wines Using GC-QTOF-MS and Metabolic Profiling Methods

**DOI:** 10.3390/molecules23092376

**Published:** 2018-09-17

**Authors:** Fang Yuan, Ke Cheng, Jihui Gao, Siyi Pan

**Affiliations:** 1 College of Food Science and Technology, Huazhong Agricultural University, Wuhan 430070, China; kecheng1234@outlook.com (K.C.); jihuigao@outlook.com (J.G.); pansiyi@mail.hzau.edu.cn (S.P.); 2 Key Laboratory of Environment Correlative Dietology (Huazhong Agricultural University), Ministry of Education, Wuhan 430070, China

**Keywords:** blueberry wine, volatile composition, multivariate analysis, GC-QTOF-MS analysis

## Abstract

A non-targeted volatile metabolomic approach based on the gas chromatography-quadrupole time of fight-mass spectrometry (GC-QTOF-MS) coupled with two different sample extraction techniques (solid phase extraction and solid phase microextraction) was developed. Combined mass spectra of blueberry wine samples, which originated from two different cultivars, were subjected to orthogonal partial least squares-discriminant analysis (OPLS-DA). Principal component analysis (PCA) reveals an excellent separation and OPLS-DA highlight metabolic features responsible for the separation. Metabolic features responsible for the observed separation were tentatively assigned to phenylethyl alcohol, cinnamyl alcohol, benzenepropanol, 3-hydroxy-benzenethanol, methyl eugenol, methyl isoeugenol, (E)-asarone, (Z)-asarone, and terpenes. Several of the selected markers enabled a distinction in secondary metabolism to be drawn between two blueberry cultivars. It highlights the metabolomic approaches to find out the influence of blueberry cultivar on a volatile composition in a complex blueberry wine matrix. The distinction in secondary metabolism indicated a possible O-methyltransferases activity difference among the two cultivars.

## 1. Introduction

Blueberries are known to be a potential source of natural antioxidants such as anthocyanins [1] and phenolics [2], and have demonstrated a broad spectrum of biomedical functions [3,4,5]. Blueberries are widely grown around the world, and their production in China has grown every year since being introduced from United States in 1989 [6]. Blueberry wine is a berry fruit wine that has dark red color, pleasant blueberry aroma, and may have a multitude of health benefits [7,8]. Although not as famous as grape wine, blueberry wine is quickly growing in popularity. The production process closely mimics that of both red and white wines. The health-enhancing antioxidants, total phenols, anthocyanins, and flavonoids in blueberry wines as well as in blueberry wine pomace have been widely reported [7,8,9,10]. But fewer studies have focused on the aroma/volatile composition of blueberry wines, although aroma is one of the most important qualities for wine products [11]. 

It is well known that grape varietal difference largely affect the aroma of corresponding wines [12,13,14]. Among various *Vaccinium* species and blueberry cultivars, significant variation in number and quantity of volatile compounds has been reported [15]. Generally, the dominant volatiles in blueberries are C_6_ compounds, such as (*E*)-2-hexenol and (*E*)-2-hexenal, followed by terpenoids and esters [16,17]. Understanding the aroma, especially cultivar aroma of blueberry wines, could help the raw material selection and lead to better quality control in blueberry wine production. However, volatile analysis in wine is always challenging, due to compound complexity, detection limit, and matrix interferences, thus it often requires multiple extraction steps. Among the various sampling techniques, solid phase extraction (SPE) is widely used in wine volatile analysis [18,19]. Among the numerous SPE phases offered today, LiChrolut-EN (ethylvinyl benzene-divinylbenzene), which was introduced in the market in the 1990s, has a high extraction capacity due to its high specific areas. It has been demonstrated that LiChrolut-EN has a much stronger retention of volatiles than other commonly used sorbents, such as bond Elut ENV (styrene- divinylbenzene), Amberlite, and Tenax TA [20,21]. Another commonly used technique is solid phase microextraction (SPME), which can extract volatile and semi-volatile organic compounds from environmental, biological, and food samples [22,23,24]. There are also a lot of commercially available stationary phases, although the divinylbenenze/carboxen/polydimethylsiloxane (DVB/CAR/PDMS) is the most frequently used on an expanded range of analytes.

Gas chromatography-mass spectrometry (GC-MS) is the most widely used technique for volatile compound detection. Limitations of the traditional GC-MS technique include its relatively low sensitivity and limited number of compound examinations. The relative concentrations of volatile compounds in foods can vary from millimolar to picomolar level, which easily exceed the linear range of the analytical techniques employed. Compounds at low concentrations, which may be of great importance, are often ignored, thus causing inconsistency and bias in the results. Recent developments in plant metabolomic techniques allow faster and more sensitive metabolite detection, which make it possible to compare complex sample matrix reliably and to identify differences and similarities objectively [25]. The metabolomic techniques can help resolve many issues and questions related to food safety, traceability, quality, new foods, transgenic foods, functional foods, nutraceuticals, etc. [26]. For example, Fourier transform ion cyclotron resonance-mass spectrometry (FTICR-MS) combined with ultra-high performance liquid chromatography-quadrupole time-of-flight mass spectrometry (UPLC-Q-TOF-MS) and multivariate statistical tools could provide a fine description of the chemical complexity and geographic origins of wines [27]. Wine metabolomic data from GC-MS could be correlated with the sensory properties of wine [28]. The application of wine metabolomics could also help to reveal new compounds in wines [29]. Besides on wine, the metabolomic techniques have been applied on many other food matrices such as tea [30], essential oils [31], as well as fermented strawberry products [32]. 

Since a huge amount of data is usually obtained from omics studies, it is necessary to develop strategies to convert the complex raw data obtained into useful information. However, most of the current approaches were focused on the non-volatile metabolites [33,34], which could not reflect the aroma quality of the sample. Therefore, in the present study, we developed a non-targeted volatile metabolomic approaches based on the GC-QTOF-MS coupled with two different sample extraction techniques (SPE and SPME), followed by multivariate statistics, to study the difference of cultivar volatile metabolites in wines made from two southern highbush blueberry cultivars (interspecific hybrids of *Vaccinium virgatum*, *Vaccinium corymbosum*, and *Vaccinium darrowii*)—“Misty” and “O’Neal”, grown in central China. Results of the analysis highlight the potential of the use of combined volatile extraction methods and metabolic tools for a direct analysis of the raw material difference of food after the complicated processing steps.

## 2. Material and Methods

### 2.1. Fruit Harvest and Winemaking

Fruits were purchased from a commercial blueberry orchard in Huangpi, Hubei, China (N 31°06′, E 114°28′). Blueberries from plants of the O’Neal and Misty cultivars were randomly harvested on the same day in June of 2016. Only berries that were at their commercial maturity were selected. The two cultivars were grown under similar horticultural conditions (e.g., irrigation, fertilization, etc.). After harvest, blueberries were cooled in an air-conditioned room (~20 °C) for half an hour, then transported to the laboratory and frozen (−20 °C, 50 h) before winemaking. Three kg of blueberries of each cultivar were thawed and crushed manually in microscale fermenters (5 L). Each cultivar was well mixed before winemaking and separated into 4 replicates to avoid compositional variation. The brix of the blueberry was measured using a PAL-1 pocket refractometer (Atago USA, Inc., Bellevue, WA, USA), and was adjusted to 20 by adding sucrose. Fifty mg/L SO_2_ (as potassium metabisulfite) was added to each ferment, and 0.02 g/kg pectinase EX (Lallemand, Montreal, QC, Canada) were added half an hour later. Fermenters were placed in a temperature-controlled rooms set at 27 °C, warmed to room temperature, and inoculated with *Saccharomyces cerevisiae* D254 (Lallemand, Montreal, QC, Canada) at approximately 10^6^ cfu/mL after rehydration, according to the manufacturer’s specifications. After 24 h of fermentation, diammonium phosphate (DAP, 300 mg/L) was added to each ferment to assist the yeast growth. During fermentation, the ferments were punched down every 24 h. After all fermentations reached dryness (<0.5 g/L reducing sugar as measured by a glucose meter, Sinocare Inc. Changsha, China), they were pressed using a cheese cloth. Wines were placed in a cold room at 14 °C to settle for 10 days, then racked, and an addition of SO_2_ (30 mg/L) was added prior to being bottled in 500 mL wine bottles and stored at 4 °C before analysis. 

### 2.2. Chemicals

LiChrolut EN cartridges (500 mg, 6 mL) were obtained from Merck (Darmstadt, Germany). All chemicals were of analytical reagent grade unless otherwise stated, and water was obtained from a Milli-Q purification system (MilliporeSigma, Burlington, MA, USA). Folin-Ciocalteu reagent, sodium carbonate, sodium acetate, potassium chloride, sodium chloride, sucrose, methanol (HPLC grade), ethanol (HPLC grade), dichloromethane (HPLC grade) and gallic acid were purchased from SCR^®^ (Shanghai, China). Eucalyptol (99%), linalool (≥95%), (−)-myrtenol (95%), carveol (97%, mixture of isomers), borneol, (≥99.0%, sum of enantiomers, GC), terpinolene (≥94.0%), β-citronellol (95%), geraniol (98%), ethyl-2-methylbutyrate (99%), ethyl-3-methylbutyrate (98%), *(Z)*-3-hexenol (98%), *(E)*-2-hexenol (96%), benzyl alcohol (≥99%), phenylethyl alcohol (≥99%), cuminic alcohol (97%), methyl butanoate (99%), isobutyl acetate (99%), ethyl butanoate (99%), isoamyl acetate (≥99%), methyl benzoate (99%), ethyl benzoate (≥99%), diethyl succinate (99%), methyl salicylate (≥99%), ethyl octanoate (≥99%), ethyl decanoate (98%), methyl vanillate (99%), decanoic acid (≥98.0%), benzaldehyde (≥99%), p-cresol (99%), 4-vinylguaiacol (≥98.0%), vanillin (99%), *(E)*-asarone (98%), *(Z)*-asarone (70%) were obtained from Sigma-Aldrich (St. Louis, MO, USA). All volatile standards were prepared by dilution with HPLC grade methanol.

### 2.3. Basic Parameter Measurements for Blueberries and Resulting Wines

The basic chemical parameters for berries and wines are shown in Table 1. The wine alcohol contents were measured by hydrometer after distillation and pH was measured by pH meter. Yeast assimilable nitrogen (YAN) of the must is expressed as the sum of primary amino nitrogen and ammonia nitrogen. The primary amino acids in blueberries were tested using an Ortho-phthaldialdehyde/N-acetyl-L-cysteine (OPA/NAC) spectrophotometric assay [35]. The ammonia nitrogen was measured using an Ammonia Assay Kit (Sigma-Aldrich, St. Louis, MO, USA). 

### 2.4. Qualitative Analysis of Aroma Compounds 

#### 2.4.1. SPE-GC-QTOF-MS

The volatiles in wine were extracted by solid phase extraction (SPE) with LiChrolut EN cartridge (500 mg, Merck, Darmstadt, Germany). The blueberry wine sample (100 mL) was diluted with 100 mL of milli-Q water, and filtrated with filter paper (medium flow, Aoke, Hangzhou, China). An aliquot of 40 mL filtered wine was passed through the LiChrolut EN cartridge, followed by 10 mL of milli-Q water. The cartridge was dried by passing through the air for 5 min, and the volatiles were eluted with 5 mL of dichloromethane. The residual water in the eluate was carefully removed using a glass dropper. The vial was then capped and stored at −20 °C until analysis. The extraction was performed in duplicate for each of the eight biological replicates (four replicates × two varieties).

A 7200 accurate-mass GC–QTOF-MS instrument (Agilent Technologies, Santa Clara, CA, USA) operated in electron impact ionization (EI) mode at 70 eV. MassHunter Acquisition B.06 was used for the determination of volatile compounds. The GC separation was performed using a fused silica HP-5MS (5% Phenyl Methyl Siloxane, 30 m × 250 μm × 0.25 μm) column. The GC oven temperature was programmed starting at 40 °C for 5 min, and increased to 180 °C at 3 °C/min and held for 1 min, then increased to 300 °C at 30 °C/min and held for 2 min. The samples were injected by an ALS autosampler (Agilent Technologies, Santa Clara, CA, USA). Splitless injections of 1 μL of sample (eluate from SPE) were carried out at 250 °C and ultra-pure grade helium was used as the carrier gas at flow rate of 1.2 mL/min. The interface and ion source temperatures were set to 300 °C and 250 °C, respectively. A solvent delay of 4 min was used to prevent damage in the ion source filament. QTOF-MS was operated at mass range of *m*/*z* 35 to 350. Mass calibration was performed daily. The in-batch order of all samples analyzed in this study was randomized with one blank sample injection for every 5 samples.

#### 2.4.2. SPME-GC-QTOF-MS

Free form volatiles in the blueberry wines were measured using the headspace-solid phase microextraction (HS-SPME) method coupled with GC-QTOF-MS. A 50/30 µm DVB/CAR/PDMS fiber (Supelco Inc., Bellefonte, PA, USA) was used for volatile extraction. One mL of wine sample was diluted with 9 mL of citrate buffer (0.2 M, pH 5.0) in a 20 mL vial, and 3 g of NaCl were added with a small magnetic stir bar. The vial was tightly capped and equilibrated at 50 °C in a thermostatic bath for 15 min and extracted by SPME fiber for 45 min at the same temperature with stirring (500 rpm). After extraction, the fiber was inserted into the injection port of GC (250 °C) to desorb the analytes. The extraction and desorption was conducted manually. The GC-QTOF-MS conditions were the same as described above, except the split ratio was 1:10, and no solvent delay was used. The extraction was performed in duplicate for each of the eight biological replicates (four replicates × two varieties). The in-batch order of all samples analyzed in this study was randomized with one blank sample injection for every 5 samples.

#### 2.4.3. Compound Identification

**Figure 1:**
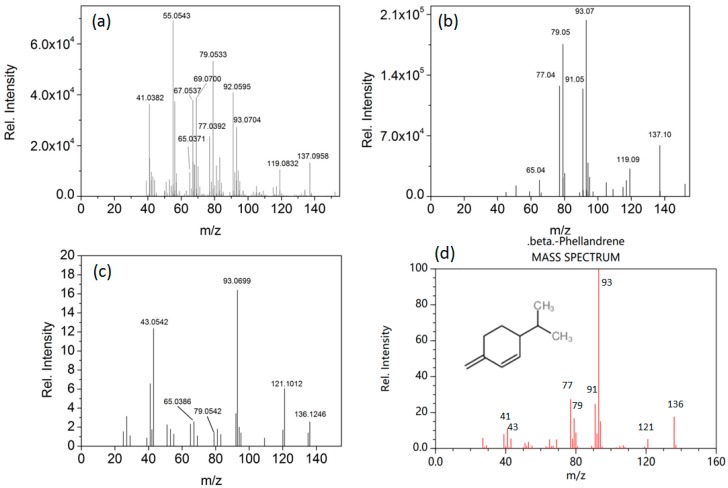
Example of β-Phellandrene detection in a blueberry wine sample (**a**) The average spectrum of β-Phellandrene extracted by conventional manual background subtraction; (**b**) the deconvoluted spectrum of β-Phellandrene. (**c**) β-Phellandrene spectrum from NIST library; (**d**) Predicted GC-MS Spectrum-GC-MS (Non-derivatized)-70 eV, Positive (HMDB0036081), obtained from The Human Metabolome Database (HMDB).

Background subtraction was first performed using the MassHunter B.06.00 software. Metabolite identification was performed manually by comparing retention times and accurate mass spectra (mass difference of less than 5 ppm and two ions) to those of the standards, when available. Tentative annotation of the chromatographic peaks, without a standard, was made by using spectral features (mass difference of less than 5 ppm theoretical value, at least one indicative fragment and isotopic pattern), literature information about chromatographic properties, mass spectra records from external databases such as The National Institute of Standards and Technology (NIST), The Human Metabolome Database (HMDB), MassBank and an internal database for the wine volatiles based on the literature, and by comparing the Kovats retention indices (RI) in NIST database and published literatures. Deconvolution supported by MassHunter B.06.00 software was performed to extract possible components in a peak if no match was found. For example, β-phellandrene was detected at 18.75 min. The average spectrum of the peak at 18.75 min extracted by conventional manual background subtraction (Figure 1a) could not match with any known compound in the library, due to the co-elution. After deconvolution, ions like *m*/*z* 93.0704, 79.0533, and 65.0371, etc., having similar peak shapes at 18.75 min, were grouped to reconstruct a deconvoluted spectrum (Figure 1b), which could be tentatively identified as β-phellandrene by comparing the spectrum with the NIST library (Figure 1c) and by comparing the accurate mass of the ions from the predicted spectrum (Figure 1d). The compound was further confirmed by comparing the calculated RI with the RI in the published literature (Table 2). 

#### 2.4.4. GC-QTOF-MS Data Pre-Processing

Data of individual GC-QTOF-MS runs were first analyzed using the MZmine 2.28 software (Free Software Foundation, Inc., Boston, MA, USA) for feature extraction, baseline correction, noise reduction, smoothing, deconvolution, grouping, and alignment according to Pluskal et al. [36]. MZmine 2.28 data processing was limited to the first 47 min of the chromatography to avoid possible interferences in the last 13 min (column bleeding and non-volatile compounds). Peak intensities of analytical replicates were averaged after peak alignment. The peak table output of MZmine 2.28 was then used for the following statistical analysis.

#### 2.4.5. Multivariate Data Analyses and Visualization

Statistical analysis was performed with the online software MetaboAnalyst version 3.0 (http://www.metaboanalyst.ca) [37]. The metabolite feature was defined as mass-to-charge ratio/retention time pair (*m*/*z*_RT pair). The principal component analysis (PCA) plots were used to obtain an overview of the large datasets and visualize similarities and metabolite features responsible for the observed patterns. The orthogonal partial least squares discriminant analysis (OPLS-DA) was performed to obtain information on differences in the volatile metabolite composition of blueberry wine samples. Markers for the difference between Misty blueberry wine (MBW) and O’Neal blueberry wine (OBW) were subsequently identified by analyzing the S-plot, which was declared with covariance (p) and correlation (p(corr)). All mass peaks were pre-processed by normalization by the median, generalized log transformation (glog2), and using a Pareto scaling for both PCA and OPLS-DA. 

## 3. Results 

### 3.1. Extraction Methods

Two volatile extraction methods, SPE and SPME, were employed in this study. SPE resulted in a higher baseline and higher noises in the mass spectrum. Compared to SPE, headspace sampling using SPME showed good sensitivity when coupled with GC-QTOF-MS, as the peaks were saturated in splitless mode in our preliminary tests. So, the split ratio was changed to 1:10 for SPME-GC-QTOF-MS later, which made the peaks sharper in shape (Figure S1, supplementary figure). SPME also resulted in a cleaner baseline, which was expected because SPME only sampled the volatiles in the headspace and no solvent was injected. The relative peak areas (Table 2 and Figure S1) show that SPME favored adsorption of low molecular weight volatiles. SPE showed a good performance on the semi-volatile compound extraction, but resulted in omission of many highly volatile compounds due to the solvent delay as well as the loss during extraction. Nevertheless, both extraction methods introduced interferences from the handling materials, such as naphthalene and dibutyl phthalate in the chromatograph. For SPME, more contaminants were found mainly due to the siloxane peaks from the fiber coating. These peaks were excluded in the identification results. 

### 3.2. Compound Identification 

Instead of directly going to statistical analysis, compound identification was first performed to get an overview of the compounds in samples and to get rid of the interference peaks/features as much as possible. We found the identification challenging, even with the well-established MS library, since a small molecule possesses the same or similar fragment after electron ionization, and a base ion was often missing, which makes it difficult to determine the structure. Many volatile compounds share similar structures, such as (*Z*)-3-hexenol and (*E*)-2-hexenol, or possess isomers, like (*Z*)-isoeugenol and (*E*)-isoeugenol, which often resulted in highly identical mass spectra. So, RI value could serve as a second, independent parameter for library matching for compound identification. A summary of volatiles from two blueberry wines is shown in Table 2. A total of 41 compounds were found using SPE-GC-QTOF-MS, while 53 compounds were observed by SPME-GC-QTOF-MS. Seventy of these volatile and semi-volatile compounds were identified or tentatively identified by using the techniques listed in the Table 2. There were thirteen alcohols, nineteen esters, one ketone, four acids, twelve terpenes, two thiols, six phenols and phenol derivatives, five norisoprenoids and eight miscellaneous compounds. Three unknown peaks were included in Table 2 since they were selected by statistical analysis as important markers, among which unknown 1 and unknown 2 showed very similar mass spectra (Figure S2, supplementary figure). However, they could not be identified by searching the MS library. 

### 3.3. Principal Component Analysis

**Figure 2:**
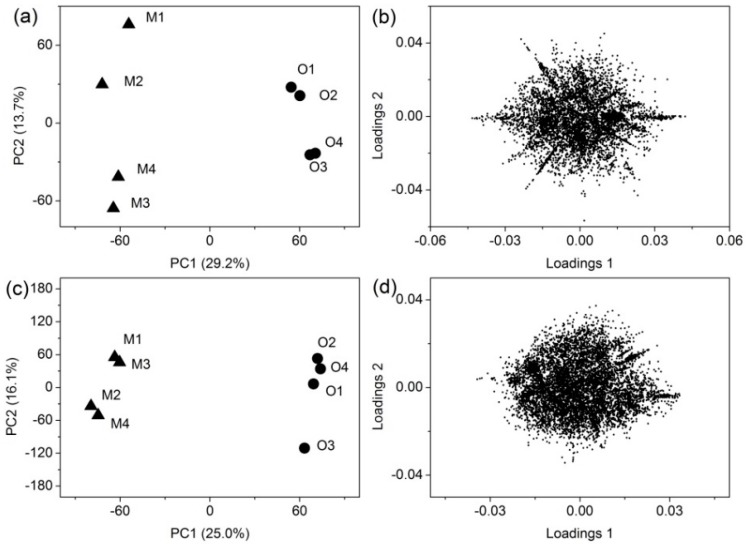
PCA of the metabolite features detected in the blueberry wines by different sample extraction method (SPE and SPME) followed by GC-QTOF-MS. The explained variances are shown in brackets. (**a**) Score plot of SPE; (**b**) Loading plot of SPE; (**c**) Score plot of SPME; (**d**) Loading plot of SPME. The PCA showing that the volatile metabolites are clearly different between Misty blueberry wine (M1, M2, M3, M4) and O’Neal blueberry wine OBW (O1, O2, O3, O4) despite of the extraction methods.

The non-targeted QTOF-MS analysis generates a tremendous amount of data and requires pre-treatment prior to the application of statistical tools. After data pre-processing, 8867 features (*m*/*z*_RT pairs) were detected by the SPE method and 12,694 features were detected by the SPME method. The principal task of the present study was the discrimination of volatile compounds between two blueberry wines, and the detection of corresponding markers. For the initial overview of the dataset, PCA was carried out. The score plots showed that both SPE and SPME methods could well distinguish the two blueberry wines, and two blueberry wines were mainly separated on principal component 1 (PC1). Score plot of SPE (Figure 2a) explained 42.9% of the total variance. Score plot of SPME (Figure 2c) explained 41.1% of the total variance. However, due to the extremely large number of data included, it was very hard to extract effective information from the loading plot of PCA (Figure 2b,d), so further statistical analysis was required. Nevertheless, the PCA results certified that the scaling method used was appropriate and there was a real separation between the two groups, as the separation was seen despite no class data being included in the algorithm. 

### 3.4. Marker Detection and Annotation

**Figure 3:**
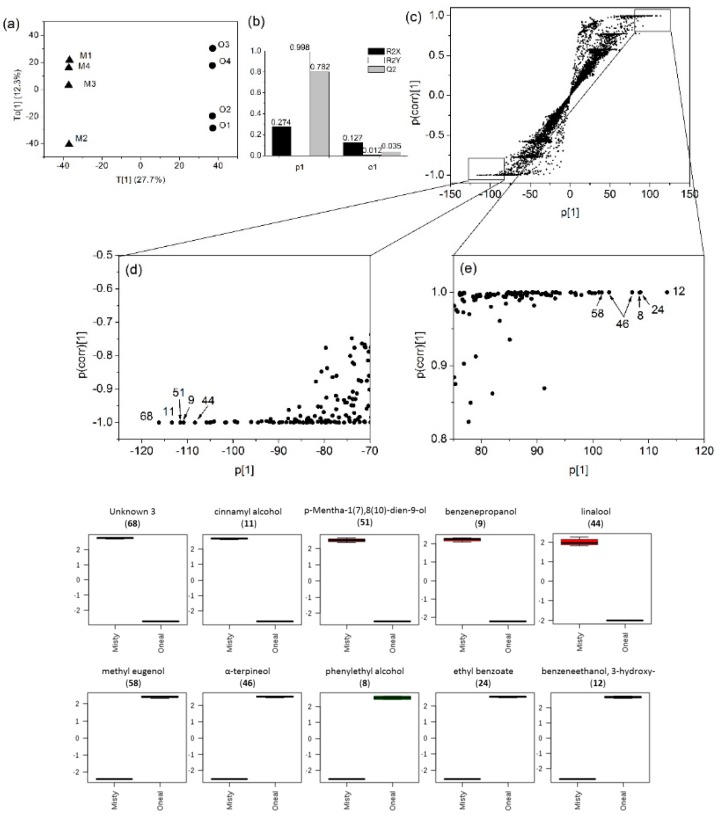
OPLS-DA of metabolite features detected in the blueberry wines by SPE-GC-QTOF-MS. (**a**) Score plot of all metabolite features; (**b**) Model overview of the OPLS-DA model; (**c**–**e**) Loadings S-plot showing the variable importance in a model, combining the covariance and the correlation (p(corr)) loading profile. The box-plots at bottom showed the significantly different volatile metabolites between Misty blueberry wine (M1, M2, M3, M4) and O’Neal blueberry wine (O1, O2, O3, O4) in the OPLS-DA model (Line, mean; box, standard error; whisker, standard deviation).

**Figure 4:**
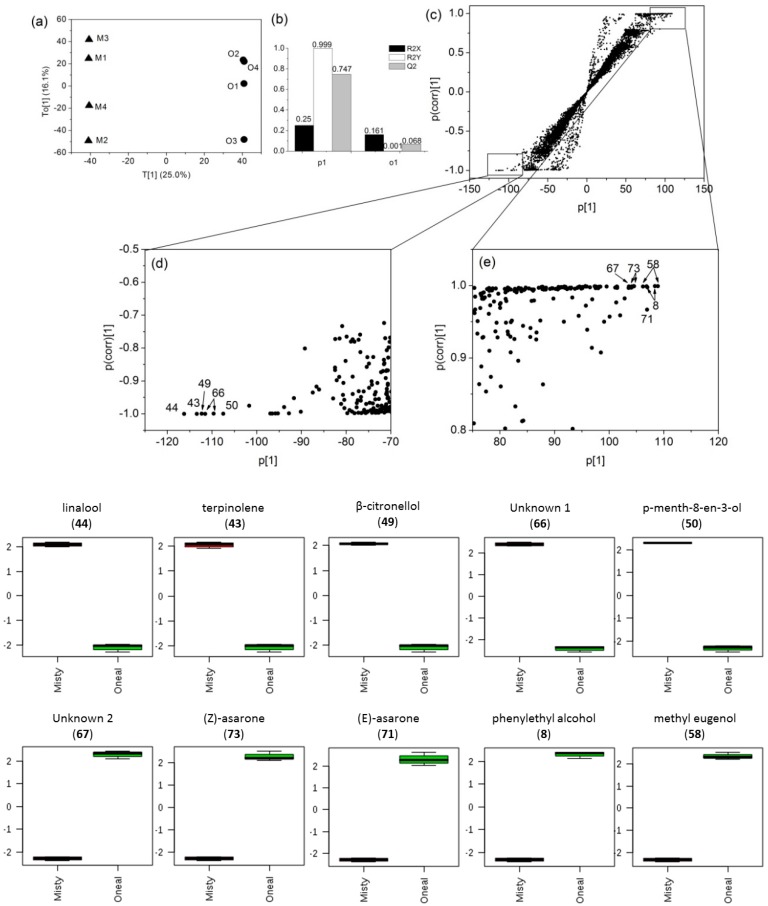
OPLS-DA of metabolite features detected in the blueberry wines by SPME-GC-QTOF-MS. (**a**) Score plot of all metabolite features; (**b**) Model overview of the OPLS-DA model; (**c**–**e**) Loadings S-plot showing the variable importance in a model, combining the covariance and the correlation (p(corr)) loading profile. The box-plots at bottom showed the significantly different volatile metabolites between Misty blueberry wine (M1, M2, M3, M4) and O’Neal blueberry wine (O1, O2, O3, O4) in the OPLS-DA model (Line, mean; box, standard error; whisker, standard deviation).

To determine possible differences between the volatile metabolite fingerprint of MBW and OBW samples, the volatile composition of two blueberry wines extracted by different methods were compared using OPLS-DA (Figure 3 and Figure 4). These models constructed with a dataset from each extraction method separate MBW from OBW along the first discriminating component (T [1]). The model showed one orthogonal component, with R^2^X = 0.274 (total variation in X explained by the model), R^2^Y = 0.998 (total variation in Y explained by the model) and Q^2^ = 0.782 (goodness of prediction) from the SPE dataset, R^2^X = 0.25, R^2^Y = 0.999 and Q^2^ = 0.747 from the SPME dataset (Figure 3b and Figure 4b), indicated that both models were validated. Potential markers for separation by different extraction methods were subsequently identified using S-plots, which were represented with covariance (p) against correlation (p(corr)). The S-plots of the OPLS-DA were proposed for the identification of potential markers of group separation by Wiklund et al. [38]. It shows the most relevant variables on the differentiation of two samples. The 10 identified markers with the highest variable influence on projection (VIP) scores by OPLS-DA are summarized in the boxplots in Figure 3 and Figure 4, with their corresponding compound no. in Table 2. The results showed that some different markers were selected by SPE and SPME. The mass peak intensities of cinnamyl alcohol (11), p-mentha-1(7),8(10)-dien-9-ol (51), benzenepropanol (9), linalool (44), methyl eugenol (58), α-terpineol (46), phenylethyl alcohol (8), ethyl benzoate (24), 3-hydroxy benzeneethanol (12) and an unknown peak (68) were significantly different between MBW and OBW samples by SPE (Figure 3). While, the mass peak intensities of linalool (11), terpinolene (43), β-citronellol (49), p-menth-8-en-3-ol (50), (*Z*)-asarone (73), (*E*)-asarone (71), phenylethyl alcohol (8), methyl eugenol (58), and two unknown peaks (66, 67) were significantly different between MBW and OBW samples by SPME (Figure 4).

## 4. Discussion 

It is generally accepted that volatile compounds influence the overall aroma profile when their concentration is above their odor thresholds. However, even when the concentration is below the odor threshold, some volatile compounds can interact with other volatiles to enhance or suppress the aroma perception [39]. Furthermore, some of them are only present at very low concentrations but often contribute greatly to the overall aroma [40]. Thus, it is important to have analytical tools suitable to detect these odor compounds, to learn the complexity behind wine aroma and to be used for selection and quality control [41]. Performances of different volatile extraction methods has been compared in many studies [41,42]. Andujar-Ortiz et al. [42] reported that SPE using a LiChrolut-EN cartridge showed very good linearity, covering a wide range of concentrations of wine volatile compounds, compared to the HS-SPME procedure. The advantage of SPME is that it can detect the highly volatile compounds which are often covered by the solvent peaks, and can eliminate problems associated with chemically and thermally unstable samples, thus avoiding generation of artifacts [43]. It has to be mentioned that the performance of the analytical methods are not only dependent on the compound extraction, but also depend on many other factors, such as column selection and instrument conditions, which are less feasible to change in real practice. In order to recover a wider range of volatile compounds in the blueberry wines, both SPME and SPE methods were used in our study. For SPE, LiChrolut-EN resins were selected because previous studies showed that they had an excellent ability for the extraction of aroma compounds from wine [42,44]. For SPME, triphase SPME fiber (DVB/CAR/PDMS) was chosen because it had been found to extract the representative blueberry volatiles [15]. The results showed a good coverage of compounds with different polarity, as well as a good coverage from the highly volatile compounds to semi-volatile compounds. The PCA plots showed that the wines made from OBW and MBW were clearly separated by the PC1, indicating the metabolomic analysis based on different extraction methods is useful to reveal the compositional differences of volatile compounds. However, we also observed different projections of the four replicates between SPE and SPME within the same variety. Misty blueberry wines in Figure 2a are projected in opposite quadrants along the PC1, showing that differences between replicates exist. The differences between replicates might come from multiple sources, since several variables were not easy to control, such as the inner temperature of fermenter, pre-fermentation extraction, as well as the yeast growth [45]. It was also interesting to note that the replicates of MBW were much closer in Figure 2c compared to Figure 2a, indicating the two extraction methods extracted different compounds thus could affect the sample being distinguished. 

Significant differences were observed between MBW and OBW samples in terms of composition and amount of the volatile compounds. However, due to the different selectivity of SPE and SPME, OPLS-DA found different markers from the two datasets, which could be complementary to each other. Linalool, methyl eugenol, and phenylethyl alcohol were selected as significantly different volatile metabolites between MBW and OBW in both of the OPLS-DA models. Only one of the fermentation-derived compounds (phenylethyl alcohol) was selected as marker using the SPME dataset, while five (phenylethyl alcohol, cinnamyl alcohol, ethyl benzoate, 3-hydroxy-benzeneethanol and benzenepropanol) were selected using the SPE dataset. It was possibly due to the different selectivity of the extraction methods, since SPME showed very poor affinity to benzenepropanol and 3-hydroxy-benzeneethanol as well as cinnamyl alcohol. The mass peak intensities of cinnamyl alcohol and benzenepropanol were higher in the MBW, while ethyl benzoate, phenylethyl alcohol, and 3-hydroxy-benzeneethanol were higher in the OBW. Among them, phenylethyl alcohol was often reported as a potential aroma compound in wine, which has a rose-like aroma [46,47]. 

The profile of fermentation-derived compounds, including alcohols, esters, acids, ketones, and volatile thiols identified in the blueberry wine were similar with those reported in the grape wines. Higher alcohols and esters mainly contribute to the fruity aroma of wine. They can be synthesized by yeast through anabolic pathway from glucose, or catabolic pathway from their corresponding amino acids [48]. As a result, their production in wine is highly dependent on the yeast stain, must YAN, fermentation temperature, and oxygen availability [49]. Since the yeast strain, DAP addition, and fermentation condition were the same in the blueberry winemaking, the differences in fermentation-derived aroma was probably associated with the different YAN of the two cultivars (Table 1), which was also a part of the cultivar characteristics. It was interesting that these fermentation derived compounds were all volatile molecules with the structure of a benzene ring, which might be associated with the metabolization of aromatic amino acids, such as tyrosine, phenylalanine, or tryptophan during fermentation [50,51], indicating the possible differences of aromatic amino acid composition of the two blueberry varieties. 

Generally, more of the important features detected in OPLS-DA were berry-derived compounds such as linalool, which has the notes of rosy and fresh fruit, and has a very strong organoleptic contribution in blueberries [15]. Besides linalool, many other terpenes were also selected by either one of the two OPLS-DA models, such as α-terpineol, p-mentha-1(7),8(10)-dien-9-ol, terpinolene, β-citronellol, and p-menth-9-en-3-ol, which was in agreement with previous report that terpenes were important aroma compounds in highbush blueberries and their concentration varied with cultivar [11,15,52]. 

Interestingly, we found that OBW contained several berry-derived semi-volatiles such as methyl eugenol, methyl isoeugenol, (*E*)-asarone, and (*Z*)-asarone, which did not exist in MBW (Table 2). Methyl eugenol and methyl isoeugenol are isomers, which are naturally occurring flavors and fragrances found in a variety of different food sources, including spices (nutmeg, allspice), herbs (basil, tarragon), and fruit, including bananas and oranges [53,54]. Methyl eugenol is also one of the plant metabolites used for defense against herbivores and pathogens as well as attracting pollinators [54]. Eugenol, isoeugenol, methyl eugenol, and methyl isoeugenol share the initial biosynthetic steps with the lignin biochemical pathway [55]. Eugenol and isoeugenol can undergo further methylation and require O-methyltransferases for the downstream production of methyl eugenol and methyl isoeugenol [56]. In the present study, eugenol and isoeugenol were found in both blueberry wines, but methyl eugenol and methyl isoeugenol were only found in OBW, indicating the possible O-methyltransferases activity difference among the two cultivars, which needs to be confirmed in the future studies. *(Z)*-Asarone has been found in glycoside form in pineapple wines [57]. It has also been reported as bioactive compounds in some of the medicinal plants such as rhizomes of *Acorus gramineus* [58]. It is interesting that *(Z)*-asarone has been reported to have neuroprotective and cardiovascular protective effects in an animal model [59]. Compared to the well-known antioxidants in blueberry wines, such as anthocyanins and phenolics, these compounds have received much less attention and might be worth to be further explored. 

The other group of volatile compounds that was very different among the two blueberry wine samples was the C_13_-norisoprenoids. C_13_-norisoprenoids are degradation products of carotenoids in many plants including blueberry [60,61]. Many of them are also well-known scent compounds with extremely low sensory thresholds, and are also important sources of grape-derived flavors in wines [62]. Among the five C_13_-norisoprenoids tentatively identified, only 4-(2,2,4-trimethylcyclohex-3-enyl) but-3-en-2-one was found in both blueberry wines. Dihydro-β-ionol was only detected in OBW, while β-ionol, 3-hydroxy-7,8-dihydro-β-ionol, and 4-(2,6,6-trimethyl-1,3-cyclohexadien-1-yl)-2-butanone were only observed in MBW, indicating that the two cultivars might differ in the C_13_-norisoprenods biosynthesis pathway. Although the related gene expression has been reported in blueberry species [61], little information was found for the C_13_-norisoprenoids metabolites in blueberries as well as blueberry wine. Du & Rouseff [15] tentatively identified that β-damascenone was an odor active compound in southern highbush blueberries by SPME-GC-olfactometry analysis, but no peak was found, possibly due to low concentration. Our results showed that various C_13_-norisoprenoids were present in the blueberry wines, although further study is still needed to confirm their sensory contributions. 

The use of SPE/SPME and GC-QTOF-MS for non-targeted volatile metabolic profiling and metabolite identification of blueberry wine was shown here. It provided good group separation and revealed possible markers for O’Neal and Misty blueberry wines, several of which were unknown to date. Further studies of these compounds in blueberries could help to confirm their cultivar correlation. The use of combined volatile extraction methods provided a significant advantage to such approaches since more complete volatile profiles were recovered. The results revealed the applicability of this approach in non-targeted studies of volatile compounds of blueberry wines and possibly other complex food products. 

## Figures and Tables

**Table 1:** Basic parameters for fruit and wine samples in this study. Mean ± SD presented (n = 4).

Sample	Parameters	O’Neal	Misty
Berry	TSS	11.4 ± 0.4	11.2 ± 0.3
	pH	3.33 ± 0.03	3.25 ± 0.06
	Berry Water Content (%)	86 ± 0	87 ± 0
	Berry Density (g/cm3)	1.0 ± 0.0	1.0 ± 0.0
	Yeast Assimilable Nitrogen (mg/L)	138 ± 2	204 ± 5
Wine	Alcohol Content (%)	10.5 ± 0.2	9.8 ± 0.1
	pH	3.05 ± 0.02	2.94 ± 0.03

**Table 2:** Characterization and relative peak size ^a^ of the volatile compounds detected in different blueberry wines by different volatile extraction methods.

No.	Compound	Exact Mass (Da)	RI ^b^	LRI ^c^	Identification ^d^	SPE		SPME	
						Misty	O’Neal	Misty	O’Neal
	**Alcohols**								
1	ethanol	46.042	730	668	MS ^e^, RIL ^f^			+++	+++
2	1-pentanol	88.089	771	766	MS, RIL			+++	+++
3	2-Methylbutan-1-ol	88.089	773	779	MS, RIL			t ^i^	t
4	(*Z*)-3-hexenol	86.073	856	858	S ^g^, MS, RI ^h^			+	+
5	(*E*)-2-hexenol	86.073	865	853	S, MS, RIL			t	t
6	2-ethyl-1-hexanol	130.136	1025	1032	MS, RIL	+	+		
7	benzyl alcohol	108.058	1029	1039	S, MS, RIL	++	+	+	+
8	phenylethyl alcohol	122.073	1111	1111	S, MS, RIL	+++	+++	+++	+++
9	benzenepropanol	136.089	1227	1231	MS, RIL	++	t		
10	cuminic alcohol	150.104	1286	1284	S, MS, RIL			+	++
11	cinnamyl alcohol	134.073	1301	1312	MS, RIL	++	t	t	t
12	3-hydroxy-benzeneethanol	138.068	1422	-	MS	++	+++		
13	homovanillyl alcohol	168.079	1527	1530	MS, RIL	+	+		
	**Esters**								
14	ethyl acetate	88.052	742	628	S, MS, RI			+++	+++
15	methyl butanoate	102.068	768	724	S, MS, RIL			+	+
16	isobutyl acetate	116.084	794	776	S, MS, RIL			+	+
17	methyl isovalerate	116.084	795	765	MS, RIL			+	++
18	ethyl butanoate	116.084	811	804	S, MS, RIL			+	+
19	ethyl 2-methylbutanoate	130.099	851	849	S, MS, RI			+	+
20	ethyl 3-methylbutanoate	130.099	854	853	MS, RI			+	++
21	isoamyl acetate	130.099	875	876	S, MS, RIL			++	++
22	ethyl hexanoate	144.115	998	1002	MS, RIL	+	+	+	+
23	methyl benzoate	136.052	1090	1103	S, MS, RIL			++	++
24	ethyl benzoate	150.068	1168	1185	S, MS, RIL	+	++	++	+++
25	phenylethyl formate	150.068	1178	1176	MS, RIL			+	+
26	diethyl succinate	174.089	1183	1167	S, MS, RIL	++	+	+++	+++
27	methyl salicylate	152.047	1189	1198	S, MS, RIL			+	++
28	ethyl octanoate	172.146	1198	1198	S, MS, RIL	++	+	+++	++
29	ethyl decanoate	200.178	1395	1398	S, MS, RIL			+	+
30	methyl vanillate	182.058	1513	1525	S, MS, RIL	++	+		
31	ethyl 4-hydroxyphenylacetate	180.079	1550	1559	MS, RIL	+	t		
32	benzoic acid, 3,4,5-trimethoxy-, methyl ester	226.084	1718	-	MS		+		
	**Ketones**								
33	3-hydroxy-2-butanone	88.0524	742	718	MS, RIL			+++	+++
	**Acids**								
34	isovaleric acid	102.068	865	877	MS, RIL	++	+		
35	hexanoic acid	116.084	991	982	MS, RIL	+	+		
36	decanoic acid	172.146	1368	1373	S, MS, RIL	+	+	+	+
37	homovanillic acid	182.058	1639	1633	MS, RIL	+	+		
	**Aldehyde**								
38	benzaldehyde	106.042	953	960	S, MS, RIL			+	+++
39	2,4-dimethyl benzaldehyde	134.073	1208	1181	MS, RIL			+	+
40	syringaldehyde	182.058	1653	1667	MS, RIL	t	t		
	**Terpenes**								
41	eucalyptol	154.136	1024	1030	S, MS, RI			+	t
42	β-phellandrene	136.125	1068	1053	MS, RIL			+	t
43	terpinolene	136.125	1098	1087	S, MS, RI	+	+	+++	++
44	linalool	154.136	1099	1096	S, MS, RI	++	++	+++	++
45	borneol	154.136	1160	1162	S, MS, RI			+	++
46	α-terpineol	154.136	1187	1186	S, MS, RI	+	++	+	++
47	myrtenol	152.120	1191	1194	S, MS, RI			+	++
48	(*E*)-carveol	152.121	1216	1217	S, MS, RI	+	+	+	+
49	β-citronellol	156.151	1125	1233	S, MS, RI			++	+
50	p-menth-8-en-3-ol	154.136	1336	-	MS	+	t	t	t
51	p-mentha-1(7),8(10)-dien-9-ol	152.120	1340	-	MS	++	+		
52	(*E*)-sobrerol	170.131	1374	1374	MS, RIL	+	t		
	**Thiols**								
53	methionol	135.230	978	978	MS, RIL	++	+		
54	dihydro-2-methyl-3(2H)-thiophenone	116.030	985	994	MS, RIL	+	+	+	+
	**Phenols and derivatives**								
55	p-cresol	108.058	1075	1075	S, MS, RIL			t	++
56	4-vinylguaiacol	150.068	1309	1323	S,MS, RIL			+	++
57	eugenol	164.084	1354	1355	S, MS, RI	+	+	++	+++
58	methyl eugenol	178.099	1402	1404	MS, RIL		++		+
59	(*Z*)- or (*E*)-isoeugenol	164.084	1445	1438/1454	MS, RIL	+	+	+	+
60	methyl isoeugenol	178.099	1495	1492	MS, RIL		+		+
	**Norisoprenoids**								
61	4-(2,2,4-trimethylcyclohex-3-enyl)but-3-en-2-one	192.151	1216	-	MS			+	+
62	β-ionol	194.167	1426	1428	MS, RIL	+			
63	4-(2,6,6-trimethyl-1,3-cyclohexadien-1-yl)-2-butanone	196.183	1428	1424	MS, RIL	+			
64	dihydro-β-ionol	196.183	1442	1449	MS, RIL				+
65	3-hydroxy-7,8-dihydro-β-ionol	208.146	1686	1683	MS, RIL	+			
	**Miscellaneous**								
66	unknown 1	-	1227	-		+	+	++	+
67	unknown 2	-	1253	-				+	++
68	unknown 3	-	1359	-		+++	+		
69	vanillin	152.047	1392	1410	S, MS, RIL	t	t	+	+
70	acetovanillone	166.063	1480	1490	MS, RIL	+	+	t	t
71	(*E*)-asarone	208.110	1556	1561	S, MS, RIL		+++		+
72	2,6-dimethoxybenzoquinone	168.042	1561	-	MS	+	+		
73	(*Z*)-asarone	208.110	1649	1646	S, MS, RIL		+++		+

^a^ The relative peak size was presented. +, the TIC peak area was less than 2 × 10^6^; ++, the TIC peak area was between 2 × 10^6^ and 5 × 10^7^; +++, the TIC peak area was larger than 5 × 10^7^. ^b^ Kovats index calculated on a HP-5MS capillary column; ^c^ Kovats index reported in published literatures; ^d^ Identification method; ^e^ Mass spectrum matched with NIST library; ^f^ Calculated Kovats index matched with NIST database or published literature; ^g^ Compound identified by comparing mass spectrum with authentic standards; ^h^ Compound identified by comparing the retention time with authentic standards; ^i^ Trace level, no peak but target ions can be extracted from background.

## Supplementary Materials

Supplementary materials are available online.

## Abbreviations

SPE	solid phase extraction
SPME	solid phase microextraction
GC-QTOF-MS	gas chromatography-quadrupole time of fight-mass spectrometry
RI	Kovats retention index
MS	mass spectrum
PCA	principal component analysis
OPLS-DA	orthogonal partial least squares discriminant analysis
MBW	Misty blueberry wine; OBW, O’Neal blueberry wine
