# Supplementary material for: Characterization of Cultivar Differences of Blueberry Wines Using GC-QTOF-MS and Metabolic Profiling Methods
Source: Molecules. 2018 Sep 17;23(9):2376. doi: 10.3390/molecules23092376 (PMC6225290; doi:10.3390/molecules23092376)
Supplement: Supplementary file 1 [file molecules-23-02376-s001.pdf]

Characterization of Cultivar Differences of Blueberry Wines Using GC-QTOF-MS and Metabolic Profiling Methods

Supplementary data

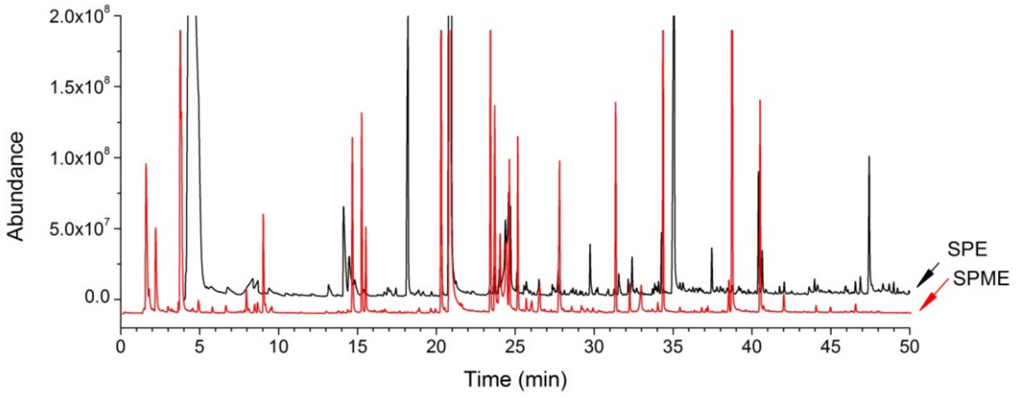

Figure S1 Example of separation and peak shape of O'Neal blueberry wine using SPE-GC-QTOF-MS and SPME-GC-QTOF-MS method.

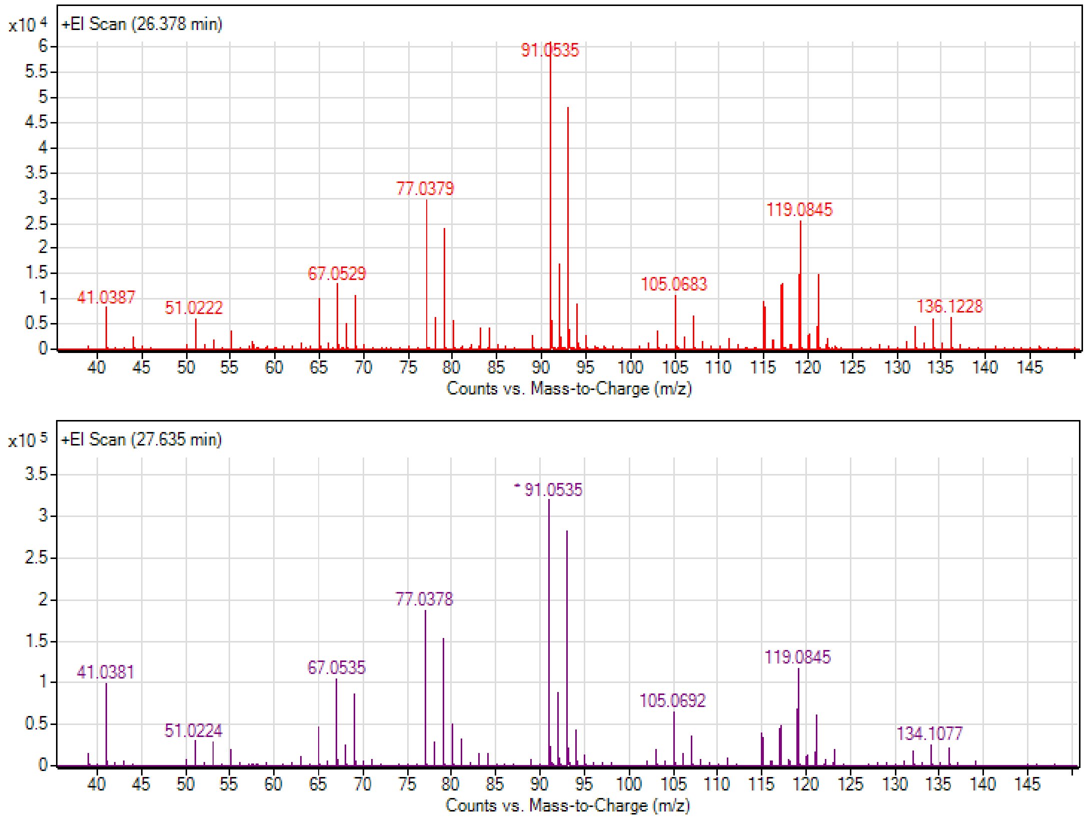

Figure S2 Mass spectra of unknown peak 1 and 2. The similar pattern indicated they were possibly isomers.
